# Ipsi- and Contralateral Oligo- and Polysynaptic Reflexes in Humans Revealed by Low-Frequency Epidural Electrical Stimulation of the Lumbar Spinal Cord

**DOI:** 10.3390/brainsci11010112

**Published:** 2021-01-16

**Authors:** Ursula S. Hofstoetter, Simon M. Danner, Brigitta Freundl, Heinrich Binder, Peter Lackner, Karen Minassian

**Affiliations:** 1 Center for Medical Physics and Biomedical Engineering, Medical University of Vienna, 1090 Vienna, Austria; ursula.hofstoetter@meduniwien.ac.at; 2 Department of Neurobiology and Anatomy, College of Medicine, Drexel University, Philadelphia, PA 19129, USA; smd395@drexel.edu; 3 Neurological Center, Klinik Penzing—Wiener Gesundheitsverbund, 1140 Vienna, Austria; brigitta.freundl@gesundheitsverbund.at (B.F.); heinrich.dr.binder@outlook.com (H.B.); peter.lackner@gesundheitsverbund.at (P.L.)

**Keywords:** commissural neurons, crossed reflexes, epidural electrical stimulation, human, locomotion, motor control, oligosynaptic reflexes, polysynaptic reflexes, spinal cord injury, spinal cord stimulation

## Abstract

Epidural electrical stimulation (EES) applied over the human lumbosacral spinal cord provides access to afferent fibers from virtually all lower-extremity nerves. These afferents connect to spinal networks that play a pivotal role in the control of locomotion. Studying EES-evoked responses mediated through these networks can identify some of their functional components. We here analyzed electromyographic (EMG) responses evoked by low-frequency (2–6 Hz) EES derived from eight individuals with chronic, motor complete spinal cord injury. We identified and separately analyzed three previously undescribed response types: first, crossed reflexes with onset latencies of ~55 ms evoked in the hamstrings; second, oligosynaptic reflexes within 50 ms post-stimulus superimposed on the monosynaptic posterior root-muscle reflexes in the flexor muscle tibialis anterior, but with higher thresholds and no rate-sensitive depression; third, polysynaptic responses with variable EMG shapes within 50–450 ms post-stimulus evoked in the tibialis anterior and triceps surae, some of which demonstrated consistent changes in latencies with graded EES. Our observations suggest the activation of commissural neurons, lumbar propriospinal interneurons, and components of the late flexion reflex circuits through group I and II proprioceptive afferent inputs. These potential neural underpinnings have all been related to spinal locomotion in experimental studies.

## 1. Introduction

A growing number of studies have demonstrated that epidural electrical stimulation (EES) can engage spinal neural circuits to enable overground walking in individuals with severe spinal cord injury (SCI) [1,2,3,4]. Common to these studies is the placement of the epidural electrodes over the lumbar and upper sacral spinal cord segments. There is a general agreement that the motor effects in the lower extremities are initiated by the electrical stimulation of local posterior roots [5,6,7,8,9]. The posterior roots carry group I and II proprioceptive afferents originating in muscles and joints of the hip and leg region [10,11,12], which presents a distinctive anatomical difference from the cervical and thoracic dorsal columns, the targets of early EES applications in motor disorders [13,14,15,16]. The group I and II proprioceptive afferents project on a neural machinery composed of motoneurons and interneurons of the gray matter, which mediate reflexes of various complexity and control functional synergies and locomotion [17,18,19,20]. In theory, EES could activate or modulate any of these circuits indirectly through the rich synaptic contacts of the stimulated afferents. Yet, the actually recruited spinal network components mediating the observed locomotor effects have largely remained unclear. To date, EES studies in individuals with SCI have revealed the elicitation of monosynaptic spinal reflexes, termed posterior root-muscle (PRM) reflexes according to their initiation and detection sites [21,22,23], the activation of premotoneuronal inhibitory circuits [24], the recruitment of a flexion synergy exploited for facilitating the swing phase for walking [1], and the generation of rhythmic activities resembling the output of central pattern generators observed in experimental animals [25,26,27]. Animal experimental studies have been pivotal in developing novel EES strategies for restoring walking after SCI [1,28,29], but have not contributed to the understanding of the motor effects of EES in humans to an equal degree. In intact and spinal rats, single-pulse EES of the lumbosacral spinal cord evoked a late response (LR) superimposed on the monosynaptic reflex (middle response, MR) [7,30,31]. The LR occurred with an additional delay of 4–5 ms, distinguishing it as an oligosynaptic spinal reflex, and was thought to involve group II muscle afferents [7] or Flexor Reflex Afferents [30,31]. There are only a few reports on the elicitation of non-monosynaptic spinal reflexes by EES in humans. The elicitation of irregular electromyographic (EMG) activities in the tibialis anterior (TA) and iliopsoas with latencies >60 ms by 2-Hz EES was observed in an individual with complete SCI [32]. Late-latency responses succeeding a monosynaptic PRM reflex in the hamstrings muscle group (Ham), evoked by 2-Hz EES in an individual with motor-complete SCI, were exemplarily shown in [6].

Here, our objective was to explore and characterize non-monosynaptic EMG responses to EES in individuals with chronic, motor-complete SCI with the aim of revealing some of the circuit components potentially mediating the motor effects in functional EES applications. EMG data from lower-extremity muscles evoked by EES at the lowest available stimulation frequencies (2–6 Hz) were analyzed to enable the investigation of responses evoked by individual pulses within continuous trains of stimuli. Using an exploratory approach, we identified three previously undescribed ipsilateral and contralateral oligo- and polysynaptic response types. We then investigated their EMG features and identified characteristic EES amplitude-dependent and rate-sensitive behaviors that were specific to each response type, which suggested candidate neural underpinnings. While these structures cannot be unequivocally unveiled in humans, our data propose the recruitment of commissural neurons, lumbar propriospinal interneurons, and components of the late flexion reflex within circuits that are associated with spinal locomotor activity.

## 2. Materials and Methods

### 2.1. Human Subjects

Data were derived from eight individuals with a median age (interquartile range; IQR) of 25.0 years (21.8–26.5 years) who had been referred to a clinical program for the treatment of lower-extremity spasticity by EES of the lumbar spinal cord [33]. All subjects had traumatic, motor-complete SCI in the chronic stage post-injury (3.4 years (2.6–4.7 years)), six classified as American Spinal Injury Association Impairment Scale (AIS) A and two as AIS B (Table 1). EMG recordings of lower-extremity muscle responses evoked by percutaneously placed, midline-located epidural linear leads were available from all subjects. Subjects 4 and 7 were further tested with additional, bilaterally placed percutaneous linear leads, with the electrodes of both leads positioned at the rostrocaudal levels of the midline leads during the trial phase, before full implantation of the epidural system. Retrospective data analysis was approved by the Ethics Committee of the City of Vienna (EK-17-059-VK).

### 2.2. Epidural Electrical Stimulation of the Spinal Cord and EMG Recordings

**Figure 1:**
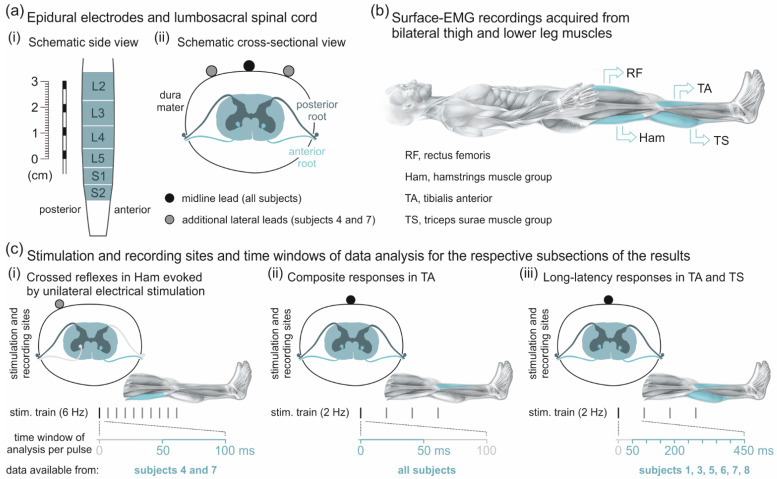
Overview of epidural electrical stimulation, EMG recording, and data analysis. (**a**) Percutaneous linear lead with four cylindrical electrodes (black rectangles) and lumbar (L) and upper sacral (S) spinal cord segments innervating lower-extremity muscles; (**i**) dimensions and (**ii**) electrode positions with respect to spinal cord and roots. (**b**) Surface-EMG locations; all recordings were conducted in the supine position. (**c**) Stimulation and recording sites, stimulation frequencies, and stimulus-triggered time windows of data analysis for the three different response types. stim., stimulus.

EES was applied through percutaneous linear leads (Model 3487A; Medtronic, Minneapolis, MN, USA) placed in the posterior epidural space (Figure 1a(i)), individually ranging from the T11 to L1 vertebral levels, and connected to an implantable pulse generator (Itrel 3, Medtronic, Minneapolis, MN, USA). Recordings 1−3 of subject 4 and recording 1 of subject 7 were conducted during the trial phase, with the medial and the additional, bilaterally placed leads (Figure 1a(ii)) externalized and connected, one at a time, to a test stimulator (3625-G, Medtronic, Minneapolis, MN, USA). The leads carried four cylindrical electrodes (referred to as 0 to 3 from rostral to caudal direction), each with a diameter of 1.3 mm, a length of 3 mm, and an inter-electrode spacing of 6 mm. Bipolar electrode combinations with different spacing between cathode (-) and anode (+) were tested, and monopolar stimulation was carried out by setting one electrode as cathode and the implantable pulse generator case as anode. Pulse width was set at 210 µs. With a given electrode setup, EES was applied at the lowest programmable frequency (implantable pulse generator: ~2 Hz; test stimulator: ~6 Hz). Stimulation amplitude was increased in 1-V increments up to a maximum of 10 V or less in case of discomfort. In subjects 3, 6, 7, and 8, higher stimulation frequencies were additionally tested, including approximately 5 Hz, 11 Hz, 16 Hz, and 21 Hz (Table 1, Frequency study). 

All EMG recordings were conducted in the supine position (Figure 1b). Pairs of silver-silver chloride surface EMG electrodes (Intec Medizinitechnik GmbH, Klagenfurt, Austria) were placed bilaterally over rectus femoris (RF), TA, Ham, and the triceps surae muscle group (TS) with a longitudinal alignment and an inter-electrode distance of 3 cm [23]. A common ground electrode was placed over the iliac crest. Abrasive paste (Nuprep, Weaver and Company, Aurora, CO, USA) was used for skin preparation to reduce EMG electrode resistance below 5 kΩ. Additional EMG electrodes placed over the lower rectus abdominis and paraspinal muscles captured stimulation artifacts used to identify the onsets of stimulation pulses. EMG signals were amplified (Grass Instruments, Quincy, MA, USA) with a gain of 2000, filtered to a bandwidth of 30–700 Hz, and digitized at 2002 samples per second and channel using a Codas analog-to-digital converter system (Dataq Instruments, Akron, OH, USA).

### 2.3. Data Analysis and Statistics

Data were analyzed using Matlab R2019a (The MathWorks, Inc., Natick, MA, USA) and NumPy 1.19.2 [34] for Python 3.8.6 (Python Software Foundation, Wilmington, DE, USA). Statistical analysis was performed with IBM SPSS Statistics 26.0 for Windows (IBM Corporation, Armonk, NY, USA) and R 4.0.0 (R Foundation for Statistical Computing, Vienna, Austria). The specific statistical tests conducted are identified in the respective paragraphs of the Methods section below. Descriptive statistics are reported as mean ± standard deviation (SD) or standard error (SE) for normally distributed data and as median and IQR for non-parametric distributions. The coefficient of variation (CoV) was calculated by dividing the standard deviation by the respective mean. Boxplots illustrate medians as bold horizontal lines within boxes spanning the IQR, and whiskers extending to the smallest and largest values that are not outliers (values 1.5–3 times the IQR; plotted as circles) or extreme values (values >3 times the IQR; asterisks). Statistical model assumptions were confirmed, residuals visually screened for normal distribution using quantile-quantile (QQ) plots, and datasets transformed if necessary (log transformations). Effect sizes were reported by the partial eta-squared ($\eta_{p}^{2}$) for linear mixed models, else by the correlation coefficient r. The strength of associations was indicated by Cramer’s V. α-errors of *p* < 0.05 were considered significant. All post-hoc tests were Bonferroni corrected. Adjusted residuals (AR) with absolute values >2 were considered to indicate significant deviations from independence.

Effective EES sites were classified into three categories based on the relative thresholds of PRM reflexes in RF and TS evoked at the lowest programmable stimulation frequencies. Threshold was defined as the lowest EES amplitude that evoked a mean EMG peak-to-peak amplitude ≥50 µV across available responses. The categories were: RF bias, threshold RF < threshold TS, indicating mid-lumbar stimulation; non-selective, threshold RF = threshold TS, lower lumbar stimulation; and TS bias, threshold RF > threshold TS, lower lumbar/upper sacral stimulation [9]. 

#### 2.3.1. Analysis of Responses to Unilateral Epidural Electrical Stimulation

EMG responses to unilateral EES at ~6 Hz in subject 4, recordings 1–3, and subject 7, recording 1, were analyzed within time windows of 0–100 ms following each pulse within the stimulus train. For each electrode setup and EES amplitude, EMG peak-to-peak amplitudes were separately calculated for PRM reflexes occurring within 0–50 ms and for crossed reflexes within 50–100 ms post-stimulus (Figure 1c(i)). Thresholds of the crossed reflexes were defined as the minimum EES amplitude evoking responses of which at least 25% had EMG peak-to-peak amplitudes ≥50 µV. Onset and offset latencies of the ipsilateral PRM reflexes and of the crossed reflexes in Ham were calculated as the first and last time points post-stimulus when the rectified EMG exceeded 10% of the peak value or 10 µV, whichever was larger. Response duration was defined as the difference between offset and onset latencies. Onset latencies and their SDs, response durations, as well as the 90th percentile of EMG peak-to-peak amplitudes and respective CoV of ipsilateral monosynaptic PRM reflexes as well as crossed reflexes in Ham were compared using separate Wilcoxon rank-sum tests. In subject 4, recordings 1–3, the impact of increasing the EES frequency from ~6 Hz to ~9 Hz was additionally investigated. To this end, mean EMG peak-to-peak amplitudes of the ipsilateral monosynaptic PRM reflexes as well as of the crossed reflexes in Ham elicited within 2–5 s before and after the change in EES frequency were determined. For both response types, the mean values obtained for the two frequencies were compared using the Wilcoxon signed-rank test. 

#### 2.3.2. Analysis of Responses in Tibialis Anterior with and without Additional Late EMG Components

**Figure A1:**
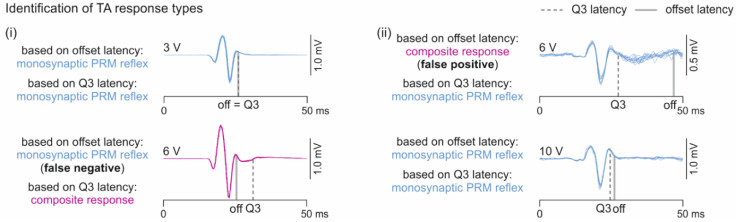
Identification of TA response types based on offset and Q3 latencies. (**i**) Response type classification based on the offset latency produced a false negative result for the responses evoked with EES at 6 V; subject 2, recording 1, electrode setup 0−3+. (**ii**) Response type classification based on the offset latency produced a false positive result for the responses evoked with EES at 6 V; subject 3, recording 1, 2−3+. In both examples, response type classification based on the Q3 latency produced correct results.

Two TA response types were distinguished, the monosynaptic PRM reflex and a composite response comprised of monosynaptic and additional late EMG components, both occurring within 0–50 ms post-stimulus (Figure 1c(ii)). Individual responses were semi-automatically assigned to one of these types by introducing the third quartile (Q3) latency. For its calculation, we determined the area under the curve of the rectified EMG signals from 5 ms to 50 ms post-stimulus to avoid stimulation artifact contamination and to fully capture all EMG potentials evoked. The Q3 latency was then defined as the time point post-stimulus with 75% of the area preceding it. We ran a k-means clustering algorithm (k = 2) on the Q3 latencies obtained for the responses of a given data set (15 repetitions with different initial points to minimize the squared distance of the clique within clusters), which then associated with their closest centroid. In case of a distance between the centroids of the two clusters of less than 3 ms, the clustering was collapsed and the responses were assigned to either the monosynaptic PRM reflex or the composite response type based on the Q3 latency values and confirmed by visual inspection of the EMG signals. The Q3 latency proved to be a robust parameter to semi-automatically identify the composite response type, while the detection based on the offset latency could lead to both false negative results (e.g., in case of large monosynaptic PRM reflex with small late EMG components; Figure A1(i) in Appendix A) as well as false positive results (Figure A1(ii)).

Response thresholds were separately identified for the monosynaptic PRM reflexes (mean peak-to-peak amplitudes ≥ 50 µV across responses) and the late EMG components of the composite responses (lowest EES amplitude at which a late EMG component was detected based on the Q3 latency) and compared using a paired Student’s t-test. To test for associations between the elicitation of composite TA responses and (i) stimulation site category (RF bias, non-selective, TS bias) as well as (ii) EES amplitudes, categorized into 1.0 × lowest TA response threshold; (1.0; 1.5] × threshold; (1.5; 2.0] × threshold; (2.0; 3.0] × threshold, and >3.0 × threshold, we conducted separate χ^2^-tests of independence. For each dataset, the proportional occurrence of the composite TA responses was identified by the number of their observations with respect to the total number of responses elicited. A Kruskal–Wallis test was run to assess for an association between the proportional occurrence and the EES amplitude category. Peak-to-peak amplitudes and corresponding CoVs were separately determined for the monosynaptic PRM reflexes and the monosynaptic as well as the late EMG components of the composite responses and compared using the Wilcoxon rank-sum test. 

Onset and offset latencies of the TA responses were defined as in the analysis of the responses evoked by unilateral EES. Major potential peaks of the EMG signals were defined as local maxima and minima separated by ≥5 ms. For the statistical analysis of the onset, offset, and Q3 latencies as well as the number of major potential peaks, the TA responses were classified into: group 1, monosynaptic PRM reflexes evoked across all tested EES amplitudes with a given electrode setup; group 2, monosynaptic PRM reflexes evoked with electrode setups that generated late EMG components only with higher stimulation amplitudes; and group 3, composite TA responses with a proportional occurrence of 1.0 (cf. Results-section). Linear mixed models with response groups as fixed factors and subject and recording as random factors were separately run on the onset, offset, and Q3 latencies as well as the numbers of major potential peaks. 

For the assessment of post-activation depression and low-frequency depression of the monosynaptic PRM reflexes and, separately, of the monosynaptic and the late EMG components of the composite responses, datasets with EES at frequencies of 2 Hz, 5 Hz, 11 Hz, 16 Hz, and 21 Hz derived from subjects 3 and 6–8, one recording each, were available (Table 1). For post-activation depression, EMG peak-to-peak amplitudes of the responses to the first two stimuli of a train were calculated and ratios of the second to the first responses were obtained. For low-frequency depression, the mean EMG peak-to-peak amplitude of the 11th–30th response within a data set was determined and normalized to the mean EMG peak-to-peak amplitude of the responses to 2-Hz EES applied with the same electrode setup and EES amplitude. Separate linear mixed models were run on the normalized response amplitudes, with interstimulus interval (post-activation depression) and EES frequency (low-frequency depression) and response type as fixed factors, and subject and recording as random factors.

#### 2.3.3. Descending Polysynaptic Responses

Responses in RF, TA, and TS to EES at ~2 Hz derived from six subjects, 13 recordings (1–4 per subject), were analyzed within time windows of 0–450 ms post-stimulus (Figure 1c(iii)). A dataset was considered if the mean EMG peak-to-peak amplitude within 50–450 ms post-stimulus was ≥50 µV and larger than within 0–50 ms post-stimulus for at least one of the applied EES amplitudes. If considered, datasets of a recording with a given electrode setup obtained with all EES amplitudes tested were included in the analysis. Activation thresholds were defined as the minimum EES amplitude at which at least 50% of the responses had peak-to-peak amplitudes ≥50 µV within the 0–450 ms time window. Then, for time windows of 0–50 ms and 50–450 ms, separate response thresholds were identified analogously and assigned to the PRM reflex and a long-latency response, respectively. PRM reflex thresholds of RF were compared to those of the TA as well as TS PRM reflexes, and the thresholds of the TA and TS PRM reflexes were compared to the thresholds of the homonymous long-latency responses using paired *t*-tests. 

EES amplitudes were separately categorized for each muscle into 1.0 × threshold; (1.0; 1.5] × threshold; (1.5; 2.0] × threshold; and >2.0 × threshold. EMG-root mean square (RMS) values were calculated for time windows of 0–50 ms, 50–100 ms, 100–150 ms, 150–200 ms and 200–450 ms. To estimate balanced mean values, a generalized linear mixed model with muscle, time window, and EES amplitude category as fixed effects was run on the EMG-RMS values. Nested random effects for subject, recording, limb (left, right), and electrode setup were included. The full-factorial dispersion model was estimated to account for heteroscedasticity; a Gamma distribution with a log-link was used. The model was fit using Template Model Builder interfaced through the glmmTMB 1.0.1 for R 4.0.0 [35]. Backwards elimination using the likelihood-ratio test was used to determine the final model.

## 3. Results

### 3.1. Crossed Reflexes in the Hamstrings Muscle Group

**Figure 2:**
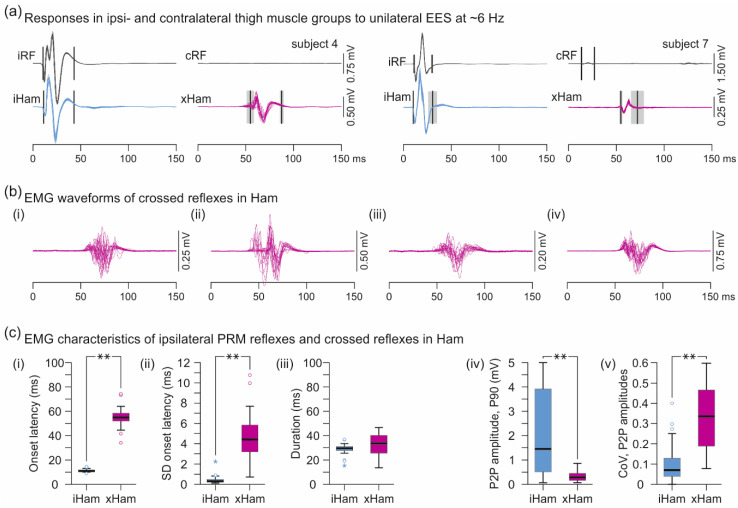
EMG characteristics of crossed reflexes in the hamstrings muscle group (Ham) evoked by unilateral EES at ~6 Hz. (**a**) EMG recordings of ipsilateral monosynaptic PRM reflexes in rectus femoris (RF) and Ham and crossed reflexes in Ham evoked by EES over the right lumbar posterior roots, subjects 4 and 7. No responses were evoked in the tibialis anterior and the triceps surae muscle group (not shown). Black vertical lines mark mean onset and offset latencies; shaded areas are SDs. (**b**) Crossed reflexes in Ham with considerable variations of EMG waveforms. All examples derived from subject 4, (**i**) and (**ii**), recording 1, left Ham, electrode setup 2+3−, 5 V and 8 V, respectively; (**iii**), recording 2, right Ham, 2+3−, 7 V; (iv), recording 3, left Ham, 1+3−, 6 V. Twenty stimulus-triggered EMG traces shown superimposed in each panel. (**c**) Boxplots of (**i**) onset latencies; (**ii**) SDs of onset latencies; (**iii**) EMG response durations; (**iv**) 90th percentiles (P90) of EMG peak-to-peak (P2P) amplitudes; and (**v**) coefficients of variation (CoV) of the EMG P2P amplitudes shown separately for the ipsilateral monosynaptic PRM reflexes and the crossed reflexes in Ham. Circles illustrate outliers and asterisks extreme values. c, contralateral; EES, epidural electrical stimulation; i, ipsilateral; PRM, posterior root-muscle; SD, standard deviation; x, crossed reflex; **, *p* < 0.001.

In subjects 4 and 7, two percutaneous linear leads were additionally placed left and right to the midline lead during the trial phase of stimulation (Figure 1a(ii)). Unilateral EES at ~6 Hz elicited ipsilateral monosynaptic PRM reflexes [22,23,24] and, in addition, a previously undescribed type of stimulus-evoked crossed reflexes in the contralateral Ham with distinctly longer onset latencies of ~55 ms (Figure 2a). In subject 4, such crossed reflexes were found in both lower extremities, while in subject 7, they occurred on the left side only. Unilateral stimulation of lumbar posterior roots was corroborated by the low thresholds of the ipsilateral monosynaptic PRM reflexes in RF and the 2.7–6.0 times higher thresholds or absence of contralateral monosynaptic PRM reflexes in the thigh muscle groups (Table 2). Thresholds of the crossed reflex normalized to the respective thresholds of the ipsilateral monosynaptic PRM reflex in Ham were, for all cases observed, subject 4, 0.75; 1.00; 1.20; and 2.50 times; and subject 7, 1.00 times (Table 2); and normalized to the respective thresholds of the ipsilateral monosynaptic PRM reflex in RF, subject 4, 0.75; 1.33; 2.00; and 5.00 times; and subject 7, 1.00 times. Notably, all electrode setups resulting in the crossed reflexes in Ham yielded lower thresholds for RF than TS PRM reflexes (Table 2). Moreover, screening of the EMG recordings showed strong co-activation of lower abdominal and paraspinal muscles (not documented here). Thus, the stimulation sites must have been located over the upper lumbar spinal cord, rostral to the L4−S2 segments containing the Ham motoneuron pool [9,36,37]. 

EMG waveforms of the crossed reflexes in Ham could resemble those of the ipsilateral monosynaptic PRM reflex (Figure 2a), but were generally more complex, exhibiting multiple peaks and considerable fluctuations when evoked repetitively with constant stimulation parameters (Figure 2b). The median onset latency (IQR) of the crossed reflexes amounted to 54.8 ms (52.9–58.4 ms) and was significantly longer than of the ipsilateral PRM reflexes in Ham, 11.0 ms (10.6–11.6 ms), z = −5.981, *p* < 0.001, r = 1.000 (Figure 2c(i)). With repeated stimulation, the onset latencies of the crossed reflexes showed apparent fluctuations and had a significantly higher SD per stimulation condition of 4.41 ms (3.19–5.83 ms) than the onset latencies of the monosynaptic PRM reflexes, 0.32 ms (0.26–0.49 ms), z = −5.981, *p* < 0.001, r = 0.984 (Figure 2c(ii)). The duration of the EMG potentials associated with the crossed reflexes showed a wider range but did not differ statistically from the ipsilateral monosynaptic PRM reflexes; crossed reflexes: 33.5 ms (25.5–40.1 ms), ipsilateral PRM reflexes: 29.5 ms (27.5–31.0 ms), z = −1.794, *p* = 0.073, r = 0.300 (Figure 2c(iii)). The maximum attainable EMG peak-to-peak amplitudes (90th percentile) of the crossed reflexes were significantly lower than of the ipsilateral PRM reflexes in Ham; crossed reflexes: 290.9 µV (161.3–452.3 µV), ipsilateral PRM reflexes: 1446.7 µV (511.1–3914.9 µV), z = 4.322, *p* < 0.001, r = 0.806 (Figure 2c(iv)), yet demonstrated higher variability when evoked with constant stimulation parameters, reflected by the CoV; crossed reflexes: 0.336 (0.188–0.466), ipsilateral PRM reflexes: 0.070 (0.039–0.130), z = −4.823, *p* < 0.001, r = 0.806 (Figure 2c(v)). 

**Figure 3:**
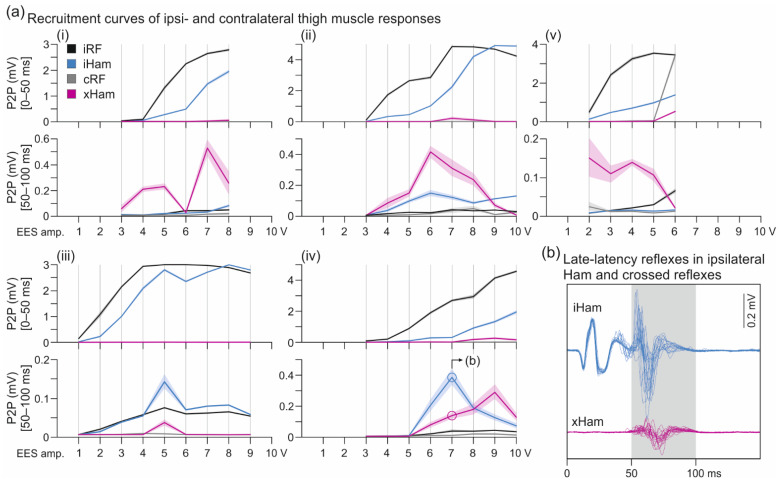
Recruitment of ipsi- and contralateral responses with graded unilateral EES at ~6 Hz. (**a**) Recruitment curves of monosynaptic posterior root-muscle reflexes of rectus femoris (RF) and the hamstrings muscle group (Ham; analysis time window: 0–50 ms post-stimulus) and of crossed reflexes (50–100 ms). All five observed cases shown in individual panels; (**i**) recording 1, right-sided EES; (**ii**) recording 2, left-sided EES; (**iii**) recording 3, left-sided EES; (**iv**) recording 3, right-sided EES; all subject 4; (**v**) recording 1, right-sided EES, subject 7. EMG analysis within the 50–100 ms time window identified late-latency responses in the ipsilateral Ham in subject 4, shown in (**b**) for recording 3, right-sided EES, 7 V. amp., amplitude; c, contralateral; EES, epidural electrical stimulation; i, ipsilateral; P2P, peak-to-peak EMG amplitude; x, crossed reflex.

The relationship between EES amplitude and EMG peak-to-peak amplitudes of responses in ipsi- and contralateral thigh muscles is illustrated for the available cases in Figure 3a, separately for time windows covering the monosynaptic PRM reflexes (0–50 ms post-stimulus) and the crossed reflexes (50–100 ms). The recruitment of the ipsilateral monosynaptic PRM reflexes in RF and Ham essentially followed a monotonous increase up to a plateau. The recruitment of the crossed reflexes in Ham was more complex, seemingly decreasing with graded EES amplitudes after having reached a maximum response size. Notably, in subject 4, recordings 2 and 3, responses within the 50–100 ms time window also occurred in the ipsilateral Ham (Figure 3a(ii–iv)) that shared similarities with the crossed reflex in terms of EMG waveforms, latencies, and the characteristic variably with repeated stimulation (Figure 3b).

**Figure 4:**
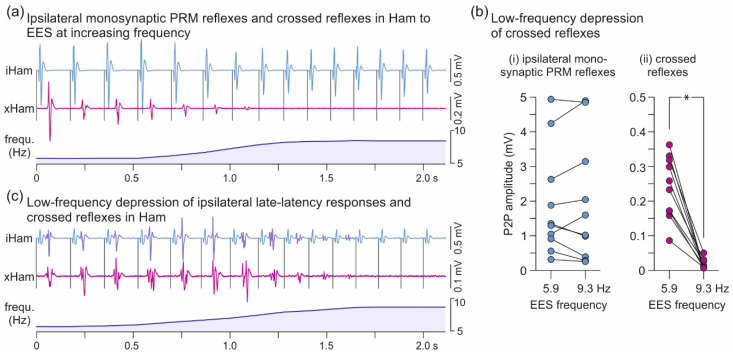
Low-frequency depression of ipsilateral Ham reflexes and crossed reflexes. (**a**) EMG recordings of ipsilateral PRM reflexes in hamstrings (iHam) and crossed reflexes (xHam) during ongoing EES, while stimulation frequency (frequ.) was increased from 5.73 Hz to 8.45 Hz; subject 4, recording 1, right-sided EES, 2+3−, 7 V. (**b**) Mean peak-to-peak amplitudes of iHam and xHam responses of all available datasets (*n* = 10; subject 4, recordings 1−3) evoked at a mean frequency of 5.86 ± 0.22 Hz and 9.33 ± 0.76 Hz, respectively. Fourteen to 65 responses before and after the change of frequency were analyzed per dataset. (**c**) EMG recordings of xHam as well as two separate responses in iHam, a monosynaptic PRM reflex and a late-latency response (dark blue potentials), the latter occurring within the time windows of xHam and showing a similar refractory behavior; subject 4, recording 2, left-sided EES, 2+3−, 7 V. EES, epidural electrical stimulation; PRM, posterior root-muscle; *, *p* < 0.05.

The crossed reflexes demonstrated low-frequency depression that was characteristically different from the behavior of the ipsilateral monosynaptic PRM reflexes in Ham. The exemplary EMG responses to unilateral EES shown in Figure 4a demonstrated complete suppression of the crossed reflexes in Ham at a frequency as low as 8.32 Hz, while the ipsilateral monosynaptic PRM reflexes were continuously evoked. Evaluation of all available datasets (*n* = 10) confirmed the suppression of the crossed reflexes from 245.6 µV (172.8–313.46 µV) at 5.86 ± 0.22 Hz to 8.7 µV (8.0–23.5 µV) at 9.33 ± 0.76 Hz, W = 0.000, *p* = 0.002, r = 1.0, while the ipsilateral PRM reflexes demonstrated no statistical changes, 1322.5 µV (944.7–2439.2 µV) and 1307.9 µV (541.5–2866.3 µV), W = 26.000, *p* = 0.922, r = 0.200 (Figure 4b). Notably, while we did not evaluate the late-latency reflexes in the ipsilateral Ham, they seemingly showed the same low-frequency depression as the crossed reflexes (Figure 4c). 

### 3.2. Oligosynaptic Reflexes in Tibialis Anterior

**Figure 5:**
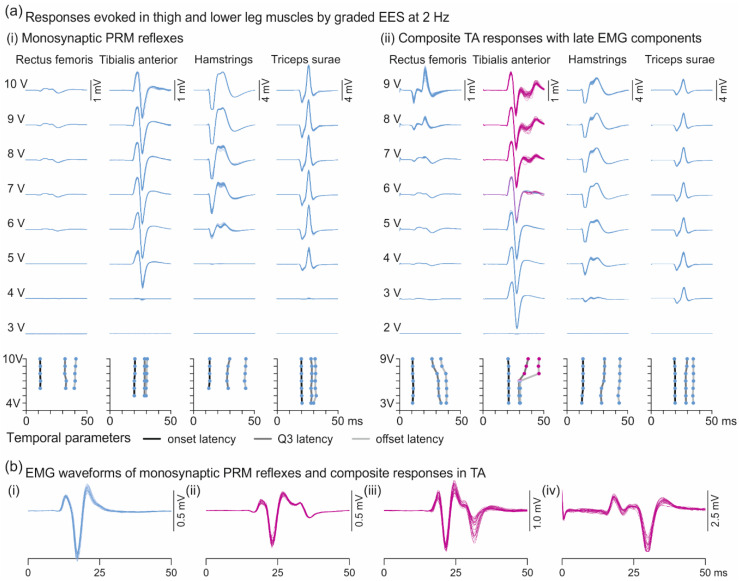
Responses of thigh and lower leg muscles to 2-Hz EES with incremental amplitudes. (**a**) (**i**) Monosynaptic PRM reflexes elicited across EES amplitudes in all four muscle groups studied. (**ii**) A change in electrode setup within the same recording resulted in the elicitation of late EMG components in TA (magenta traces) with EES amplitudes ≥2 × response threshold. Graphs below EMG traces illustrate corresponding mean onset and offset latencies as well as Q3 latencies for each EES amplitude. Note the sharp shift of offsets and Q3 latencies with the occurrence of the late EMG components. All examples derived from subject 4, recording 5, (**i**), electrode setup 0+1−, (**ii**), 1−c+. (**b**) Examples of EMG waveforms of monosynaptic PRM reflexes in TA (**i**, blue traces) and of composite TA responses (**ii**–**iv**, magenta) with increasing contribution of the late EMG components. All examples derived from subject 3, (**i**) electrode setup 0+3−, 3 V; (**ii**) 0+2−, 6 V; (**iii**) 0+3−, 5 V, all recording 2; and (**iv**) 3−c+, 10 V, recording 1. Twenty to 26 stimulus-triggered EMG traces shown superimposed in each panel. EES, epidural electrical stimulation; PRM, posterior root-muscle; TA, tibialis anterior.

A specific behavior of monosynaptic PRM reflexes is the increase in response size with graded EES amplitudes without changes in characteristic features of their EMG shapes (Figure 5a(i)). We here explored a previously undescribed response type found in TA with additional late EMG potentials superimposed on the monosynaptic PRM reflexes (Figure 5a(ii)). These composite responses had distinct polyphasic waveforms with offset latencies that remained <50 ms (Figure 5b). They were detected in all eight subjects and were elicited with 168 out of 315 (53.3%) electrode setups tested. There was a significant association between their elicitation and the stimulation-site category (RF bias, non-selective, TS bias), χ^2^(2) = 9.743, *p* = 0.008, Cramer’s V = 0.176. Non-selective stimulation sites contributed more than expected under the null hypothesis (AR, 2.1; 61.2% of all non-selective setups), while TS-bias stimulation sites contributed less than expected (AR, −2.9; 36.2%). No difference in the observation frequency between composite TA responses and monosynaptic PRM reflexes was found for RF-bias stimulation sites (AR, 0.2; 53.9%). The mean threshold ± SD of the late EMG components was significantly higher by a factor of 1.8 ± 1.2 than the TA response threshold, irrespective of the response type. Considering all electrode setups resulting in composite TA responses with at least one stimulation amplitude, mean thresholds were 3.8 ± 2.3 V for TA responses and 5.5 ± 2.3 V for the late EMG components, *t*(167) = −11.173, *p* < 0.001, r = 0.865. There was a significant association between the elicitation of the late EMG components and the EES amplitude, classified into five categories: 1.0 × TA response threshold; (1.0; 1.5] × threshold; (1.5; 2.0] × threshold; (2.0; 3.0] × threshold; and >3.0 × threshold; χ^2^(4) = 36.796, *p* < 0.001, Cramer’s V = 0.223. Stimulation at threshold contributed less (AR, −4.1) and at >3.0 × threshold more (AR, 5.0) than expected under the null hypothesis. 

**Figure 6:**
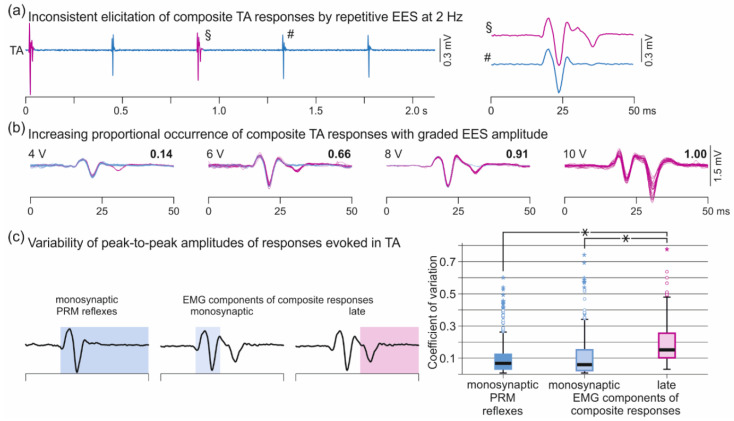
Inconsistent elicitation and variability of late EMG components in TA. (**a**) EMG recording of TA shows the elicitation of monosynaptic PRM reflexes as well as composite responses with constant stimulation parameters; subject 1, recording 1, electrode setup 0+3−, 4 V. Responses marked by § and # are illustrated in enlarged time scale on the right. (**b**) Monosynaptic (blue) and composite (magenta) responses of TA evoked with increasing EES amplitudes as indicated. Bold numbers depict proportional occurrences of composite responses. Twenty-three to 36 stimulus-triggered EMG traces shown superimposed in each panel; subject 3, recording 1, 1+3−. (**c**) Variability of EMG peak-to-peak amplitudes of monosynaptic PRM reflexes (analysis time window: response onset to 50 ms post-stimulus; *n* = 730) as well as of monosynaptic (response onset to offset of monosynaptic component) and late EMG components (offset of monosynaptic component +2 ms to 50 ms post-stimulus) of composite responses for cases with a proportional occurrence of 1 (*n* = 269). Boxplots show the distributions of the coefficient of variation of the peak-to-peak amplitudes of responses as indicated. Circles illustrate outliers and asterisks extreme values. EES, epidural electrical stimulation; PRM, posterior root-muscle; TA, tibialis anterior; *, *p* < 0.05.

TA responses with late EMG components were generally inconsistently evoked by repetitive stimulation with constant parameters (Figure 6a). Their proportional occurrence depended on the EES amplitude category, χ^2^(4) = 37.409, *p* < 0.001, r = 0.225 (Figure 6b). Post-hoc pairwise comparisons revealed a lower proportional occurrence at 1.0 × threshold compared to the (1.0; 1.5] × threshold category (*p* = 0.019); to the (2.0; 3.0] × threshold category, *p* = 0.041; and to the >3.0 × threshold category, *p* < 0.001. The proportional occurrence in the >3.0 × threshold category was significantly higher compared to any other stimulation amplitude category, all *p* < 0.001. We further examined the variability of the EMG peak-to-peak amplitudes of the monosynaptic PRM reflexes and separately for the monosynaptic and the late components of the composite responses for cases with a proportional occurrence of 1 (Figure 6c). The median CoV of the peak-to-peak amplitudes of the monosynaptic PRM reflexes was 0.068 (0.032–0.125). The median CoV of the monosynaptic components of the composite responses was 0.060 (0.023–0.256) and that of the late components 0.152 (0.102–0.256). The variability of the late but not the monosynaptic EMG components of the composite responses was significantly higher than of the monosynaptic PRM reflex (late components, z = −13.903, *p* < 0.001, r = 0.440; monosynaptic components, z = −1.182, *p* = 0.237, r = 0.037).

For the analysis of temporal parameters (Table 3), we classified the TA responses into: group 1, monosynaptic PRM reflexes evoked across all tested EES amplitudes with a given electrode setup (*n* = 728; cf. Figure 5a(i)); group 2, monosynaptic PRM reflexes evoked with electrode setups that generated late EMG components only with higher stimulation amplitudes (*n* = 262; cf. Figure 5a(ii), 3–5 V); and group 3, composite TA responses with a proportional occurrence of 1.0 (*n* = 278; cf. Figure 5a(ii), 7–9 V). Onset latencies did not differ between the three response groups, F(2;73.597) = 0.186, *p* = 0.831, $\eta_{p}^{2}$ = 0.005. For both the offset and the Q3 latency, response group was a significant factor, F(2;38.608) = 28.871, *p* < 0.001, $\eta_{p}^{2}$ = 0.599; and F(2;39.329) = 66.311, *p* < 0.001, $\eta_{p}^{2}$ = 0.771, respectively. Post-hoc pairwise comparisons revealed significantly longer offset and Q3 latencies in group 3 compared to the two other groups (all *p* < 0.001). We additionally analyzed the number of major peaks of the EMG potentials and identified response group as significant factor, F(2;43.220) = 29.757, *p* < 0.001, $\eta_{p}^{2}$ = 0.579. Post-hoc pairwise comparisons revealed significant differences between group 3 and group 1 (mean difference: 1.30 ± 0.07, *p* < 0.001) as well as group 2 (1.20 ± 0.08, *p* < 0.001). In summary, the composite responses identified by the Q3 latency had monosynaptic onset latencies but featured additional EMG peaks and prolonged offset latencies.

**Figure A2:**
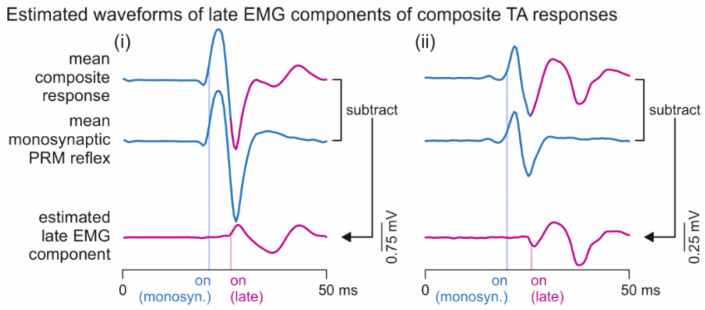
Estimated EMG waveforms of late components of composite TA responses. EMG waveforms were constructed by subtracting the averaged EMG waveform of all monosynaptic PRM reflexes from the composite responses evoked by EES with the same electrode setup and amplitude. Data derived from (**i**) subject 4, recording 5, electrode setup 0−c+, 6 V, and (**ii**) subject 7, recording 4, 0−1+, 5 V. monosyn., monosynaptic; on, onset latency; PRM, posterior root-muscle; TA, tibialis anterior.

The late EMG components were never evoked without a preceding monosynaptic PRM reflex and therefore, their onset latencies could not be directly detected in the EMG signals. We estimated their EMG signals by subtracting the averaged EMG waveforms of the monosynaptic PRM reflexes from the composite responses considering datasets with proportional occurrences of 0.1–0.9 (five subjects, 30 datasets analyzed; Figure A2). Within this subset of data, median onset latencies were 17.8 ms (16.5–19.4 ms) for the averaged monosynaptic PRM reflexes and 26.0 ms (23.6–27.9 ms) for the constructed waveforms representing the late EMG components. The onset latency of the isolated late EMG components had a delay of 8.0 ms (5.5–11.4 ms) with respect to the monosynaptic PRM reflex.

**Figure 7:**
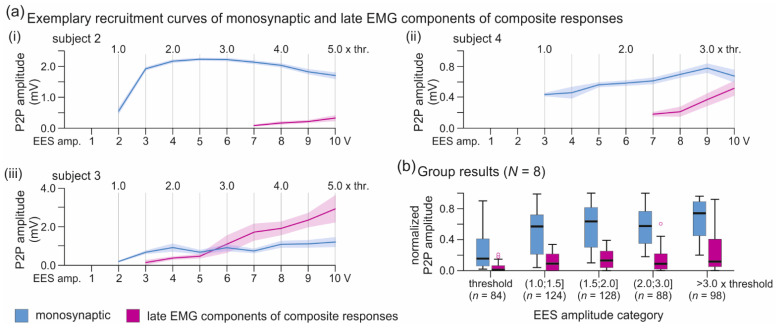
Recruitment curves of monosynaptic and late EMG components of composite TA responses with graded EES. (**a**) Individual recruitment curves of composite responses with increasing contributions of late EMG components. Data derived from (**i**) subject 2, recording 1, electrode setup 0−c+; (**ii**) subject 4, recording 5, 3−c+; (**iii**) subject 3, recording 1, 3−c+. (**b**) Group results of the recruitment of the monosynaptic and late EMG components considering cases with four or more EES amplitude levels tested. Boxplots show distributions of normalized peak-to-peak amplitudes (relative to the maximum TA responses evoked across all tested electrode setups within the same recording) for EES amplitude categories as indicated. Circles illustrate outliers. Numbers in brackets are available data per category. EES, epidural electrical stimulation; P2P, peak-to-peak; TA, tibialis anterior; thr., threshold.

We established separate recruitment curves for the monosynaptic and the late EMG components of the composite TA responses. Individual results suggested that after full recruitment of the monosynaptic reflex component, the late response could further increase with graded EES and generally did not reach a plateau with the applied EES amplitudes (Figure 7a, see also Figure 5a(ii)). Group results considering all subjects and cases with at least four EES amplitude levels tested are shown in Figure 7b.

**Figure 8:**
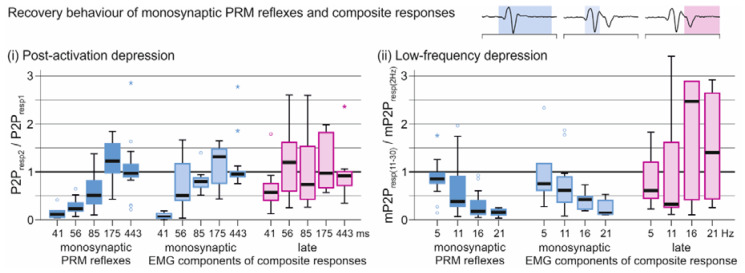
Recovery behavior of monosynaptic PRM reflexes and composite responses in tibialis anterior. (**i**) Post-activation depression of monosynaptic PRM reflexes as well as monosynaptic and late EMG components of composite responses tested by paired-pulses with increasing interstimulus intervals as indicated. The y-values are normalized peak-to-peak amplitudes (second, resp2, to first response, resp1) per interstimulus interval. (**ii**) Low-frequency depression of the respective response types tested by trains of EES with increasing stimulation frequency as indicated. The y-values are normalized mean peak-to-peak amplitudes (mean of 11th to 30th responses, resp(11–30), to mean of responses to 2 Hz, resp(2Hz)). Circles illustrate outliers and asterisks extreme values. Data derived from subjects 3, 6, 7, and 8 (one recording each); numbers of available data sets per frequency: 17–39. EES, epidural electrical stimulation; m, mean; P2P, peak-to-peak EMG amplitude; PRM, posterior root-muscle.

We investigated post-activation depression of the monosynaptic PRM reflexes and, separately, of the monosynaptic and late EMG components of the composite responses (three response categories) tested by paired pulses at interstimulus intervals of 41, 56, 85, 175, and 443 ms (Figure 8(i)). Response category, F(2;148.460) = 3.537, *p* = 0.032, $\eta_{p}^{2}$ = 0.045, and interstimulus interval, F(4;145.560) = 6.616, *p* < 0.001, $\eta_{p}^{2}$ = 0.154, were significant factors, and there was a significant interaction effect between them, F(8;148.475) = 2.078. *p* = 0.041, $\eta_{p}^{2}$ = 0.101. Post-hoc pairwise comparisons revealed significantly stronger depression of the monosynaptic PRM reflexes as well as of the monosynaptic components of the composite responses than the late EMG components (*p* = 0.001 and *p* < 0.001, respectively) at an interstimulus interval of 56 ms. Low-frequency depression of the three response types was investigated for EES frequencies of 5, 11, 16 and 21 Hz (Figure 8(ii)). There was a significant interaction between response category and EES frequency, F(6;83.561) = 2.337, *p* = 0.039, $\eta_{p}^{2}$ = 0.144. Response category, F(2;84.132) = 6.403, *p* = 0.003, $\eta_{p}^{2}$ = 0.132, and frequency, F(3;45.557) = 2.827, *p* = 0.049, $\eta_{p}^{2}$ = 0.157, were significant factors. Post-hoc comparisons revealed significantly less depression of the late EMG components of the composite responses than the monosynaptic PRM reflex (16 Hz, *p* = 0.023; 21 Hz, *p* = 0.002) as well as the monosynaptic components of the composite responses (16 Hz, *p* = 0.032; 21 Hz, *p* = 0.003) at the two highest frequencies tested. At these frequencies, the late components were rather facilitated.

### 3.3. Descending Polysynaptic Responses in Tibialis Anterior and the Triceps Surae Muscle Group

**Figure 9:**
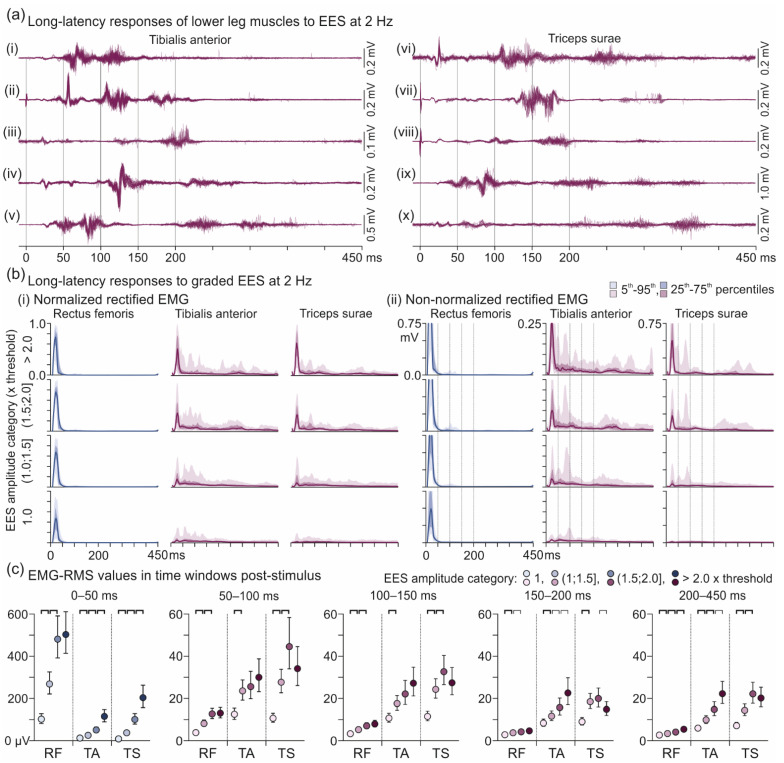
Long-latency responses in TA and TS to 2-Hz EES. (**a**) Various examples of complex, long-latency EMG activities in TA and TS evoked by EES over spinal cord segments rostral to those innervating the lower leg muscles. Shown are 19–36 stimulus-triggered traces superimposed for periods of 0–450 ms post-stimulus each. Four response time windows were defined from 0 to 200 ms and one from 200 to 450 ms. Peaks at time 0 are stimulus artifacts; evoked potentials within 0–50 ms are monosynaptic posterior root-muscle reflexes. (**i**) TA, subject 1, recording 1, 0−2+, 9 V; (**ii**) TA, subject 1, recording 3, 0−c+, 7 V; (**iii**) TA, subject 6, recording 2, 0−c+, 7 V; (**iv**) TA, subject 8, recording 2, 1+2−, 9 V; (**v**) TA, subject 8, recording 4, 0−3+ 8 V; (**vi**) TS, subject 8, recording 2, 1+2−, 9 V; (**vii**) TS, subject 3, recording 3, 0−c+, 8 V; (**viii**) TS, subject 1, recording 2, 0+1−, 8 V; (**ix**) TS, subject 8, recording 4, 0−3+, 7 V; (**x**) TS, subject 6, recording 3, 1+3−, 8 V. (**b**) Group results of rectified EMG activities of RF, TA, and TS, (**i**) normalization to the respective maximum EMG response within the same muscle and recording and (**ii**), absolute EMG values. Solid lines are median values, shaded areas with increasing opacity cover the 25th–75th and 5th–95th percentiles, respectively. Data derived from six subjects, 13 recordings. In (**ii**), monosynaptic posterior root-muscle reflexes in RF are truncated. (**c**) EMG-RMS values calculated separately for EES amplitude categories and time windows as indicated. Data points are mean values, whiskers cover the 95% confidence interval. Brackets flag significant results of post-hoc contrasts (bold lines, *p* < 0.001; thin lines, *p* < 0.05). EES, epidural electrical stimulation; RF, rectus femoris; RMS, root mean square; TA, tibialis anterior; TS, triceps surae.

2-Hz EES applied from RF-bias stimulation sites evoked long-latency responses in TA and TS, but not the thigh muscles, with variable EMG shapes and latencies within 50–450 ms post-stimulus (Figure 9a). Data derived from subjects 1, 3, 5, 6, 7, and 8, 13 recordings, were analyzed. The long-latency responses could be elicited without a preceding monosynaptic PRM reflex and occur phase-locked, as the leading activities in Figure 9a(ii,iv), or burst-like, as in Figure 9a(v,x). They frequently appeared as a close succession of two (Figure 9a(i), 50–150 ms) or three separate activations (Figure 9a(x), >200 ms). The complexity of these responses was further revealed when rectified EMG signals were averaged over all available datasets (Figure 9b). A mixed model revealed significant fixed and interaction effects of muscle (RF, TA, TS), time window (0–50 ms, 50–100 ms, 100–150 ms, 150–200 ms, and 200–450 ms), and EES amplitude category (1.0 × threshold; (1.0; 1.5] × threshold; (1.5; 2.0] × threshold; and >2.0 × threshold) on EMG-RMS values produced (all *p* < 0.001; Figure 9c). Post-hoc contrasts revealed that responses within 0–50 ms were significantly smaller in TA than RF (ratio = 0.128 ± 0.006, *t*(8282) = 45.570, *p* < 0.001) as well as in TS than RF (ratio = 0.187 ± 0.008, *t*(8282) = 36.618, *p* < 0.001). Within all other time windows, EMG-RMS values of both TA and TS were significantly larger than those of RF (all ratios >2.5, all *p* < 0.001). Further, EES amplitude category had a significant effect on the RMS-EMG values produced in all three muscles studied within each time window. Within time windows >50 ms, EMG-RMS values of TA either plateaued (50–100 ms and 100–150 ms) or increased (150–200 ms and >200 ms) with graded stimulation, while EMG-RMS values of TS plateaued (50–100 ms, 100–150 ms, and >200 ms) or decreased (150–200 ms). 

The long-latency responses were elicited in TA with 111 (62.4%) and in TS with 137 (77.0%) of the electrode setups tested. They could be elicited concomitantly in the antagonistic TA and TS (80.2% and 65.0% of all TA and TS responses, respectively) or in one of these muscles only (TA, 19.8%; TS, 35.0%). With the electrode setups leading to the long-latency TA and TS responses, mean thresholds ± SD for the elicitation of monosynaptic PRM reflexes were, RF, 5.0 ± 1.8 V, and TS, 6.9 ± 2.3 V, and were significantly different between these two muscles, *t*(136) = −18.926, *p* < 0.001. Monosynaptic PRM reflex threshold in TA was 7.3 ± 2.2 V. Thresholds of the long-latency responses were, TA, 5.3 ± 1.8 V, and TS, 4.9 ± 1.8 V, and were significantly lower than those of the respective monosynaptic PRM reflexes, TA, *t*(110) = −16.870, *p* < 0.001, and TS, *t*(136) = −22.750, *p* < 0.001. The difference between the thresholds of the long-latency TA responses and the monosynaptic PRM reflex in RF amounted to 0.3 ± 0.7 V, yet was significant, *t*(110) = −5.175, *p* < 0.001. The mean difference between the thresholds of the long-latency TS responses and the monosynaptic PRM reflex in RF was −0.1 ± 0.9 V and not significant, *t*(136) = 1.729, *p* = 0.086. 

**Figure 10:**
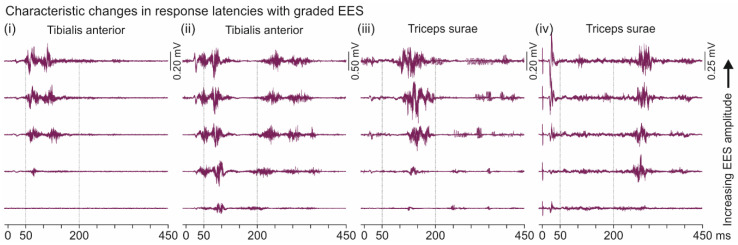
Increasing EES amplitude alters the onset of long-latency responses in TA and TS. Long-latency responses of TA (**i**,**ii**) and TS (**iii**,**iv**), ten stimulus-triggered traces superimposed each. Onset latencies of responses within 50–200 ms decreased, while those of responses within 200–450 ms increased with graded EES. (**i**) subject 1, recording 1, setup 0−2+; (**ii**) subject 8, recording 4, 0−3+; (**iii**) subject 3, recording 3, 1−3+; (**iv**) subject 8, recording 2, 3−c+; increasing EES amplitudes from bottom to top, (**i**–**iii**) 6–10 V, and (**iv**), 4–8 V. EES, epidural electrical stimulation.

While we did not attempt to classify the long-latency responses, two different types could be differentiated by their compelling behavior with graded EES (Figure 10). We identified responses within 50–200 ms, found in TA as well as in TS, which shifted to shorter onset latencies with increasing EES amplitude, and responses within 200–450 ms, which consistently showed increasing latencies. These two types could be evoked either in isolation (Figure 10(i,iii,iv)) or concomitantly (Figure 10(ii)).

## 4. Discussion

### 4.1. Crossed Reflexes in the Hamstrings Muscle Group

We propose that the contralateral Ham responses with onset latencies of ~55 ms were initiated within unilateral, upper lumbar posterior roots and were crossed reflexes. Upper lumbar stimulation sites were documented by the relative PRM reflex thresholds of the L2−L4 innervated RF and the L5−S2 innervated TS [9], together with the observed elicitation of lower trunk muscle responses. The unilateral stimulation was substantiated by the absence of contralateral monosynaptic PRM reflexes at the EES amplitudes that evoked the crossed reflexes. Given the estimated stimulation sites and the relatively low thresholds of the contralateral responses with respect to the ipsilateral PRM reflex thresholds, the most probable stimulated structures were local posterior roots [1,7], i.e., those containing afferent fibers from the femoral nerve or more rostral ones. Ipsilateral low-threshold group I as well as group II muscle spindle afferents can trans-synaptically engage spinal pathways that are crossing the spinal cord midline via commissural neurons [19,38]. Direct electrical activation of commissural neurons within the spinal cord gray matter could be excluded because of their small axonal diameters, the lack of myelin, and the fact that current penetrates the spinal cord poorly [7]. 

Remarkably, with an increase in EES frequency to ~9 Hz, the crossed reflexes were completely depressed. The absence of attenuation of the monosynaptic PRM reflexes in Ham could have been related to reduced homosynaptic depression at the synapse between group Ia muscle spindle afferents and α-motoneurons following SCI [39,40]. Homosynaptic depression has been demonstrated in other spinal pathways of group Ia afferents, as well as for group II muscle spindle afferents, but the degree of depression was shown to depend on the type of target interneurons [41,42]. Here, synaptic transmission of at least one interneuronal population specific to the crossed reflexes must have been subject to potent homosynaptic depression. While this EES frequency-dependent behavior was observed in three different recordings of subject 4, it remains to be confirmed in a larger sample of subjects. 

Recent studies in humans with intact nervous systems identified excitatory crossed actions on background EMG activity of the contralateral Ham. Ipsilateral knee flexion activating proprioceptive afferents from the quadriceps evoked a facilitatory response in the contralateral biceps femoris (BF) with an onset latency of 43.7 ± 7.2 ms during isometric contraction, thought to be induced by group II afferents [43]. Stretch of the ipsilateral Ham during treadmill walking evoked a longer-latency facilitation in the contralateral BF at 76.3 ± 6.2 ms, probably with a transcortical contribution [44]. However, neither the onset of the short-latency contralateral Ham facilitation [43] nor the role of ipsilateral Ham afferents in the generation of the longer-latency facilitation [44] are compatible with the EES-evoked crossed reflex in Ham described here. 

The rostral EES sites leading to the crossed reflexes as well as the occasional concomitant elicitation of ipsilateral Ham responses with similar latency were reminiscent of findings in animal experiments. In the cat, interneurons located in lamina VIII in the L4−L5 spinal cord segments receive monosynaptic inputs from group I and II afferents and project caudally to the contralateral motor nuclei of Ham located in the L7 segment [45]. Another population of lumbar commissural neurons with input from group I or II afferents is located in lamina VII/the intermediate zone [46]. They are predominantly excitatory, with axons projecting either contralaterally or bilaterally. In the mouse spinal cord, a major genetically identified group of excitatory glutamatergic commissural neurons are V3 interneurons [19], most of which connect with contralateral motoneurons or interneurons, while some project to both halves of the spinal cord [47]. V3 interneurons are rhythmically active during locomotion [19]. A study combining experiments in isolated spinal cord preparations of neonatal mice with computational modeling suggested that bilateral activation of V3 interneurons contributed to synchronization of left-right locomotor activity, while unilateral activation produced asymmetrical cycle frequencies, which may provide a mechanism required for walking on a curved path [48]. 

### 4.2. Oligosynaptic Reflexes in Tibialis Anterior

We propose that the late EMG components of the composite TA responses represented homonymous oligosynaptic reflexes evoked by electrical stimulation of group ll muscle spindle afferents. Compatible with the fact that group II fibers cannot be electrically recruited in isolation from group Ia, the late EMG components never occurred in the absence of a monosynaptic PRM reflex [49]. Several characteristics of the late EMG components distinguished them from the monosynaptic PRM reflexes, i.e., their higher thresholds; their further increase with graded stimulation after the monosynaptic reflex component had plateaued; their inconsistent elicitation with constant stimulation parameters; the higher variability of their peak-to-peak amplitudes; and their different behavior with repeated stimulation. 

We estimated a delay of 8.0 ms of the late EMG components with respect to the monosynaptic PRM reflex in TA, which is consistent with an oligosynaptic reflex. Previous studies had estimated the central delay of the oligosynaptic group II reflexes in humans to be longer than the monosynaptic transmission by 5.3 ms [50] and 13.0 ms [51], using different methods. The late EMG components were most readily elicited with non-selective stimulation sites previously estimated to be located over the lower lumbar spinal cord segments [9]. Hence, they were most likely homonymous reflexes, as the proprioceptive afferents of TA enter the spinal cord via the L4 and L5 roots. In cats, electrical stimulation of the L7 root, containing the proprioceptive afferents of TA in this species, was shown to evoke homonymous group II reflex discharges in the deep peroneal nerve that innervates the TA muscle [10]. Homonymous oligosynaptic group II reflexes in TA were demonstrated in humans with intact nervous system, evoked by a brisk stretch of the receptor-bearing muscle [52]. 

The composite EMG waveform of the monosynaptic and the late EMG components shared some similarities with the MR and LR evoked by single-pulse EES in hindlimb muscles in normal and spinal rats [7,30,31]. However, there were also essential electrophysiological differences. The LR had the same or lower thresholds than the MR [7,30]. Paired-pulse EES demonstrated a complete depression of both the MR and the LR for interstimulus-intervals of up to 500 ms [30], and depression by trains of stimuli was stronger in the LR [31]. These characteristics of the LR in rats do not conform to group II reflexes [49], although it was suppressed by the α2-adrenergic receptor agonist tizanidine [7], which depresses the transmission from group II but not group I muscle afferents [52,53]. Here, the monosynaptic PRM reflex and the late EMG components in TA demonstrated clear differences in their refractory behavior, both when tested with paired-pulses as well as trains of stimuli. The monosynaptic reflex was likely subject to homosynaptic depression at the higher frequencies tested [6,21,23,54,55]. The shortest excitatory synaptic linkage from group II muscle afferents to α-motoneurons described in the cat is di- or trisynaptic [49]. In the disynaptic pathway, group II afferents project on interneurons in the intermediate zone, termed group II interneurons [17], group I/II interneurons [56], or lumbar propriospinal neurons [49], which synapse on ipsilateral motoneurons. The trisynaptic pathway additionally involves dorsal horn interneurons, which excite the same interneurons in the intermediate zone intercalated in the disynaptic pathways from group II afferents [57]. Homosynaptic depression occurs at the synapse between group II afferents and the dorsal horn interneurons, but in the disynaptic pathway, it is negligible [42]. Similarly, there is no homosynaptic depression of non-monosynaptic group I transmission in humans, but rather a slight facilitation at higher stimulation frequencies [41]. There is probably a similar behavior for group II transmission to these neurons [49].

Non-monosynaptic group I and group II excitation of human lower-extremity motoneurons, including those of TA, were suggested to be relayed through rostral interneurons located in the upper lumbar spinal cord [51,58]. They presumably correspond to the lumbar propriospinal neurons interposed in the di- and trisynaptic pathways from group II afferents in the cat [59]. In the cat, they are concentrated in the midlumbar spinal cord segments, which correspond to the upper lumbar segments in humans [49]. In the paralyzed decerebrate cat, lumbar propriospinal neurons with group II input were demonstrated to be rhythmically active during fictive locomotion [60]. The neurons fired in phase with the flexor motor output measured in ipsilateral peripheral nerves, including the nerve innervating TA, while no activity was observed during extensor activity. Analogously, the oligosynaptic reflex identified here in the only monofunctional flexor muscle studied might reveal spinal network components associated with the flexor phase of locomotion in humans. There were anecdotal reports of oligosynaptic responses in TA observed in the flexor bursts of EES-induced locomotor-like activity in individuals with complete SCI during which the monosynaptic reflex components were suppressed [21,26]. Whether the oligosynaptic TA responses described here have physiological similarities with the flexor-burst related responses remains to be elucidated.

### 4.3. Descending Polysynaptic Responses in Tibialis Anterior and the Triceps Surae Muscle Group

Similar observations to which we could directly relate our findings of long-latency responses in TA and TS have not been described in literature to the best of our knowledge, hence we can only speculate about their neural underpinning. Thresholds of the long-latency responses were significantly lower than those of the monosynaptic PRM reflexes in the same muscle. At the same time, relative thresholds of PRM reflexes in RF and TS suggested that stimulation sites were located close to the L2−L4 posterior roots [9]. Further, thresholds of the long-latency responses were comparable to the monosynaptic PRM reflex in RF. Together, these data suggest that the long-latency responses in the L4-S2 innervated TA and TS could have been evoked by stimulation of the upper- or midlumbar posterior roots, including low-threshold group I proprioceptive afferents.

The long latencies could not be explained by reafferent inflow following a previous muscle contraction [61,62] as the responses could appear without a preceding monosynaptic PRM reflex and occurred in too close succession to each other. Hence, the delays could be explained by mediation through long chains of spinal interneurons. Descending heteronymous connections have been shown for group Ia muscle spindle afferents from the femoral nerve (L2−L4 segments) to the motoneuron pools of TA and the TS muscle group in humans [63,64,65]. Noteworthy, this connection links afferents from one muscle to a pair of antagonistic muscles operating at another joint [66]. A descending propriospinal system activated by group I afferents from the femoral nerve and diffusely projecting to the motoneuron pools of TA and TS has also been demonstrated in humans [58]. This relay system could be the same lumbar propriospinal neurons [49] as discussed above. Such direct or indirect projections of lumbar afferent fibers to distal motoneuron pools may also exist at interneuronal level in various reflex pathways.

A striking characteristic of two types of long-latency responses (cf. Figure 10) was the consistent change in onset latencies with increasing EES amplitude. The behavior of the responses >200 ms was reminiscent of the late flexion reflex observed in individuals with chronic SCI [67,68]. The late flexion reflex is thought to be evoked in the largest-diameter proprioceptive afferents or low-threshold cutaneous afferents, depending on the stimulated peripheral nerve. Increasing the stimulation amplitude was thought to recruit additional afferent fibers that activated an inhibitory system, delaying the onset of the late flexion reflex. Several observations had suggested that the circuits transmitting the late flexion reflex were analogous to the long-latency Flexor Reflex Afferents pathways released in the acute spinal cat after administration of DOPA [69,70], which in turn were associated with the activity of the central pattern generator in this species [71,72]. 

## 5. Conclusions

The facilitation of walking function by EES applied below a (near-) complete lesion in previous studies had suggested the recruitment of locomotor-related spinal neural circuits through proprioceptive afferent projections in humans. Here, previously undescribed lower-extremity muscle responses evoked by single pulses of low-frequency EES revealed the activation of neural circuits, all of which could be components linked to spinal locomotor control. Our data suggested the recruitment of specific pathways mediating the crossed reflex in Ham and the oligosynaptic reflex in TA, respectively, as well as of more complex sets of circuits involved in the long-latency activation of TA and TS. Common to all response types was their initiation within lumbar proprioceptive afferents and the mediation of their activity, most likely through upper lumbar interneurons, including those connecting the two halves and different segments of the spinal cord. Increasing use of EES in investigational studies will not only continue to shift the boundaries of functional recovery to levels previously deemed impossible, but will also provide new approaches to further delineate the neural mechanisms underlying locomotor control in humans.

## Figures and Tables

**Table 1:** Clinical characteristics of subject population and number of EMG recordings analyzed.

Subject No.	Gender	Age (y) ^1^	AIS Grade	Neurol. Level of SCI	SCI Chronicity (y) ^1^	No. of Recordings Analyzed		
						Lateral EES	Medial EES	Frequency Study
1	m	18.0	A	C4	2.9	0	3	0
2	m	25.6	A	C4	3.8	0	3	0
3	m	21.9	A	C7	5.1	0	3	1
4	m	21.3	A	T7	2.6	3	2	0
5	f	24.7	A	T7	4.2	0	2	0
6	m	33.2	A	T8	13.5	0	3	1
7	m	29.0	B	C6	2.5	1	3	1
8	m	25.3	B	C8	1.6	0	4	1

^1^ at the time of epidural electrode implantation; AIS, American Spinal Injury Association Impairment Scale; EES, epidural electrical stimulation; Neurol., neurological.

**Table 2:** Thresholds of ipsi- and contralateral monosynaptic PRM reflexes and crossed reflexes evoked by unilateral EES; total of five observed cases.

Subject	4				7
Recording	1	2	3		1
EES side	right	left	left	right	right
Electrode setup	2+3−	2+3−	1+3−	1+3−	1−3+
Max. EES amp. (V)	8	10	9	10	6
**Thresholds of Monosynaptic PRM Reflexes in RF and TS**					
Thr iRF (0–50 ms)	4	3	1	3	2
Thr iTS (0–50 ms)	NaN	8	3	7	5
Thr cRF (0–50 ms)	NaN	NaN	NaN	NaN	6
Thr cTS (0–50 ms)	NaN	NaN	NaN	NaN	NaN
**Thresholds of Reflexes in Ham**					
Thr iHam (0–50 ms)	4	5	2	4	2
Thr cHam (0–50 ms)	NaN	8	6	NaN	7
Thr xHam *(50–100 ms)*	3	6	5	4	2

Thresholds are given in Volts (V). c, contralateral; EES side, epidural electrical stimulation side, side of the laterally placed percutaneous lead; i, ipsilateral; Ham, hamstrings muscle group; NaN, Not a Number: no response evoked with maximum EES amplitude (Max. EES amp.); PRM, posterior root-muscle; RF, rectus femoris; Thr, threshold; TS, triceps surae muscle group; x, crossed reflex; the italics are to emphasize the difference.

**Table 3:** Temporal parameters and number of major potential peaks of monosynaptic posterior root-muscle (PRM) reflexes and composite responses in tibialis anterior.

Response Group	Onset Latency	Offset Latency	Q3 Latency	No. Peaks
1	19.61 ± 0.05 ms	31.56 ± 0.19 ms	28.55 ± 0.07 ms	2.52 ± 0.02
2	19.13 ± 0.09 ms	29.84 ± 0.30 ms	28.14 ± 0.15 ms	2.57 ± 0.04
3	18.91 ± 0.09 ms	35.31 ± 0.53 ms	33.57 ± 0.22 ms	3.47 ± 0.07

Response groups: 1, monosynaptic PRM reflexes evoked with all stimulation amplitudes tested (cf. Figure 5a(i)); 2, monosynaptic PRM reflexes developing into composite responses only with higher stimulation amplitudes (cf. Figure 5a(ii), 3–5 V); and 3, composite responses with proportional occurrences of 1.0 (cf. Figure 5a(ii), 7–9 V).

## Data Availability

The data presented in this study are available in the article.
